# Primary splenic angiosarcoma presenting with multiple bone destruction as the initial manifestation: a case report and narrative review

**DOI:** 10.3389/fonc.2026.1918735

**Published:** 2026-08-21

**Authors:** Weijun Wang, Xia Liu

**Affiliations:** 1 Medical Imaging Department of Baoji Central Hospital, Shaanxi, China; 2 Department of Ultrasound Medicine, Affiliated Baoji Hospital of Xi’an Medical university, Shaanxi, China

**Keywords:** biopsy hemorrhage, bone metastasis, differential diagnosis, imaging diagnosis, splenic angiosarcoma, vascular tumor, well-differentiated angiosarcoma

## Abstract

**Background:**

Primary splenic angiosarcoma (PSA) is an extremely rare and highly aggressive malignant vascular tumor. Its clinical presentation is often nonspecific, and the diagnosis is frequently delayed. Osseous metastasis as the initial clinical manifestation is particularly uncommon, posing significant diagnostic challenges. A critical yet underappreciated phenomenon is the discordance between histological differentiation and biological behavior in angiosarcoma: well-differentiated tumors can exhibit highly aggressive clinical courses. Furthermore, biopsy of vascular tumors carries a substantial risk of intractable hemorrhage, a complication that is often underestimated.

**Case introduction:**

We report the case of a 56-year-old female who presented with a 20-day history of lower back and right hip pain. Imaging revealed multiple osteolytic lesions involving the thoracolumbar vertebrae, pelvis, and sacrum, as well as multiple nodular lesions in the spleen. The initial clinical suspicion favored metastatic disease of unknown primary. Ultrasound-guided needle biopsy of the right iliac lesion yielded abundant hemorrhage but no definitive malignant tissue on initial pathology. Subsequent open biopsy of the L2 spinous process and right iliac bone resulted in life-threatening hemorrhage, necessitating emergency digital subtraction angiography (DSA) and super-selective embolization. Histopathological examination of the surgical specimens revealed a mixed-type vascular neoplasm with partially well-differentiated angiosarcomatous changes, with immunohistochemical positivity for CD31, CD34, ERG, and Fli-1. Remarkably, follow-up pelvic MRI performed only 21 days after the initial scan demonstrated interval enlargement of multiple bone lesions, indicating extraordinarily rapid disease progression despite the high-grade histological appearance.

**Conclusion:**

This case highlights several critical clinical considerations for angiosarcoma: (1) When both splenic and skeletal lesions coexist, suspected primary splenic angiosarcoma with bone metastases should be strongly considered; (2) The histological grade may be discordant with biological behavior—high-grade tumors can exhibit highly aggressive courses; (3) Imaging features can mimic benign vascular tumors such as hemangioma; (4) Biopsy of vascular tumors carries a substantial risk of intractable hemorrhage, and pre-procedural angiographic assessment should be considered. This case underscores the importance of integrating clinical, imaging, and pathological findings for accurate diagnosis and management of this rare malignancy.

## 1 Introduction

Angiosarcoma is a rare malignant neoplasm of endothelial cell origin, accounting for approximately 1–2% of all soft tissue sarcomas (1). It can arise in any anatomical location but most commonly affects the skin, breast, liver, and spleen. Primary splenic angiosarcoma (PSA) is exceedingly rare, with an estimated incidence of 0.14–0.23 cases per million population per year (2, 3). PSA is one of the most aggressive malignancies of the spleen, with a median survival of 5–12 months and a metastatic rate of 70–85% (3, 4).

The clinical presentation of PSA is often nonspecific, including abdominal pain, splenomegaly, fatigue, and hematological abnormalities such as anemia and thrombocytopenia (2). Osseous destruction as the initial manifestation is particularly uncommon and can lead to diagnostic confusion with primary bone tumors, hematological malignancies, or metastatic carcinoma of unknown primary (5, 6). In the 2020 World Health Organization (WHO) Classification of Soft Tissue Tumours, angiosarcoma is classified as a malignant vascular neoplasm, with morphological differentiation categorized as well-differentiated, moderately differentiated, and poorly differentiated (7). However, well-differentiated angiosarcoma can exhibit highly aggressive clinical behavior, including rapid progression and early metastasis (8, 9).

We present a case of suspected PSA presenting with multiple bone destruction as the initial clinical manifestation. This case underscores several important clinical issues: the determination of primary versus metastatic disease when both splenic and skeletal lesions coexist; the discordance between histological differentiation and biological aggressiveness; the imaging features and diagnostic challenges in differentiating angiosarcoma from benign vascular tumors; and the critical risk of hemorrhage during biopsy of vascular tumors. A comprehensive review of the relevant literature is provided.

## 2 Case presentation

A 56-year-old female patient presented with a 20-day history of lower back and right hip pain of insidious onset, without associated lower extremity numbness, radicular pain, or intermittent claudication. She initially presented at a local hospital, where lumbar computed tomography (CT) revealed multiple areas of bone destruction involving the T12 vertebral body, L1–5 vertebral bodies, L2 spinous process, and right ilium, suspicious for metastatic disease. Symptomatic treatment with oral medications was administered without clinical improvement. Ten days prior to presentation, her pain symptoms worsened, prompting referral to our institution’s Department of Orthopedics. During the course of illness, the patient remained conscious with adequate mental status, appetite, and sleep, with normal bowel and bladder function. Past medical history, personal history, marital and reproductive history, and family history were all unremarkable. Orthopedic physical examination revealed tenderness and percussion tenderness at the L4–5 vertebral level spinous processes and the right hip region; the remainder of the examination was unremarkable. Laboratory investigations revealed an elevated prothrombin time activity (PT) of 127.21% (reference range: 70–120%), a shortened prothrombin time (PT-sec) of 9.18 seconds (reference range: 10–14 seconds), a decreased international normalized ratio (PT-INR) of 0.77 (reference range: 0.8–1.2), and an elevated D-dimer of 9.73 mg/L (reference range: 0–0.5 mg/L). The coagulation profile suggested a hypercoagulable state, which is a common paraneoplastic manifestation in malignancy and may be related to the release of procoagulant substances by tumor cells. Complete blood count and the remainder of liver and renal function tests, blood biochemistry, and other laboratory investigations were unremarkable.

**Figure 1:**
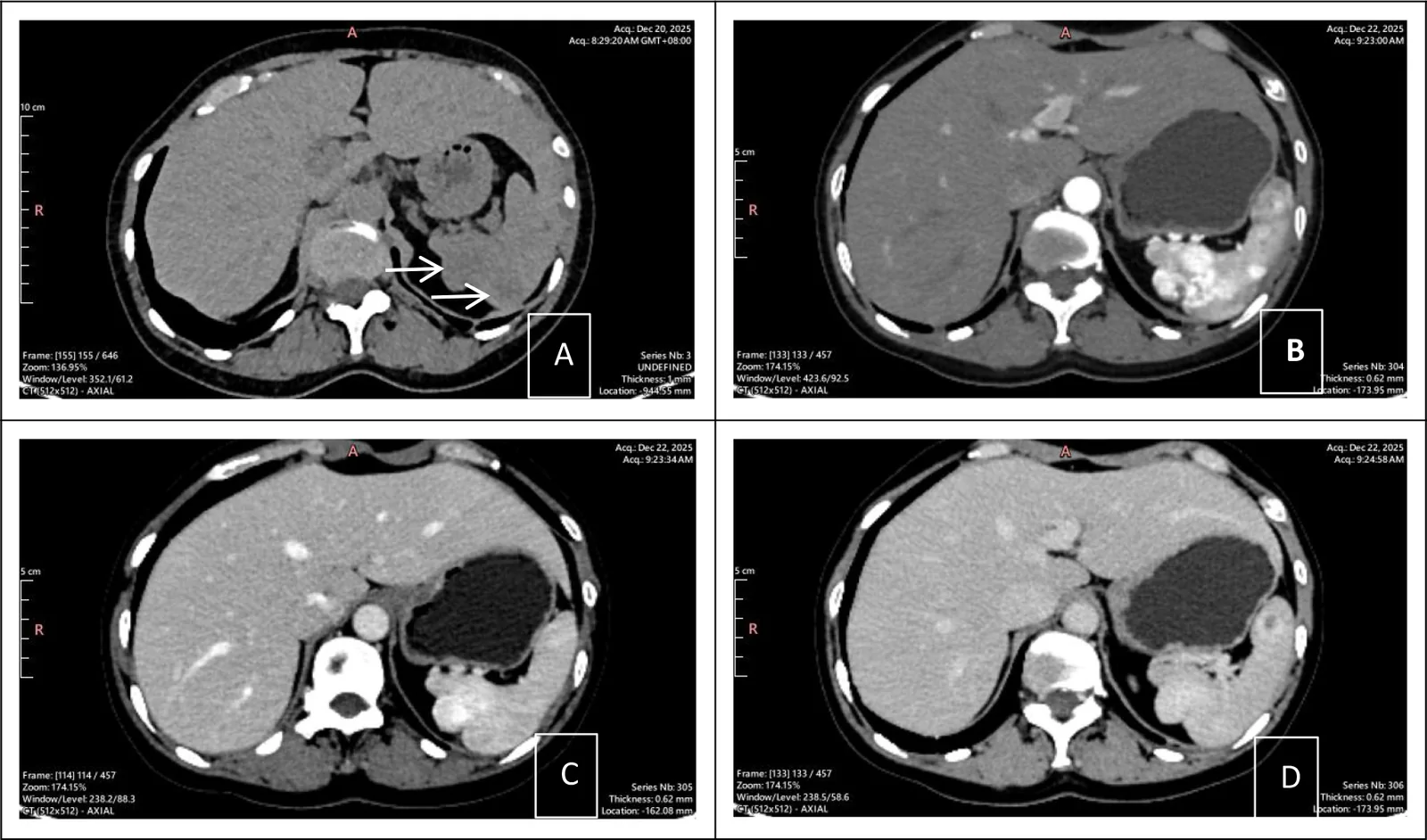
Abdominal CT imaging findings. **(A)** Non-contrast CT of the abdomen demonstrates multiple slightly low-density nodules in the spleen (arrows), some of which protrude beyond the splenic contour. **(B–D)** Contrast-enhanced abdominal CT [arterial phase **(B)**, portal venous phase **(C)**, delayed phase **(D)**] shows progressive centripetal fill-in enhancement of the splenic lesions, which gradually become isodense with the surrounding splenic parenchyma.

**Figure 2:**
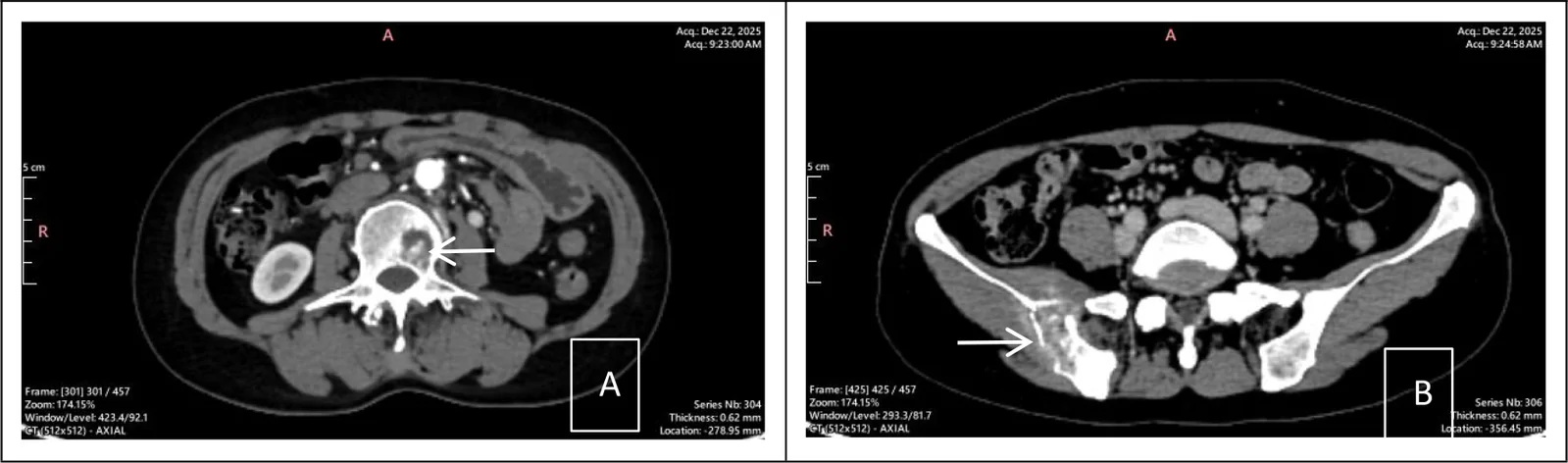
Contrast - enhanced CT imaging findings. **(A)** Heterogeneous enhancement in the bone destruction zone within the lumbar vertebral body (as indicated by arrows). **(B)** Heterogeneous enhancement in the bone destruction zone of the right ilium and the local soft tissue mass.

**Figure 3:**
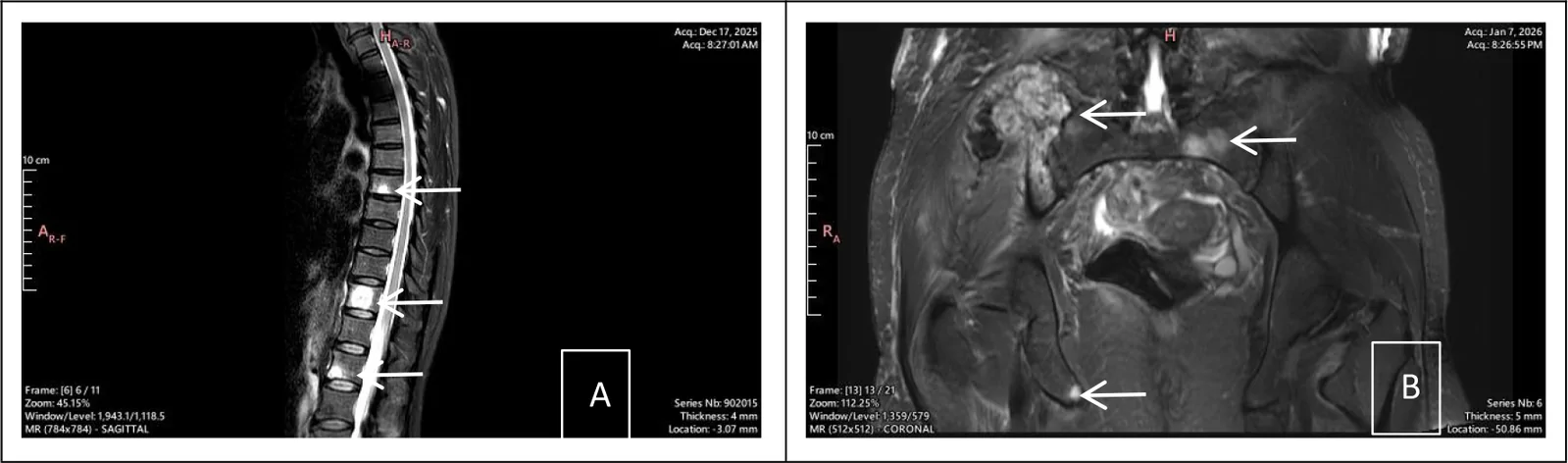
MRI findings. **(A)** Sagittal STIR of the thoracic spine, **(B)** Coronal STIR of the pelvis, showing multiple abnormal signal foci in multiple vertebral bodies and various bones of the pelvis, with a relatively large lesion in the right ilium and a surrounding soft tissue mass (as indicated by arrows).

After admission, non-contrast CT of the head, chest, abdomen, and pelvis was performed. The head CT was unremarkable. Multiple patchy low-density areas were identified within the vertebral bodies and posterior elements, as well as in the pelvic bones. The right ilium demonstrated bone destruction with cortical interruption and an associated local soft tissue mass. Multiple rounded areas of decreased density were noted within the spleen (**Figure 1A**). Contrast-enhanced abdominal CT (**Figures 1B-D**) demonstrated that the spleen was not enlarged but had an irregular contour. Multiple rounded nodular lesions were identified within the spleen, some of which protruded beyond the splenic contour. On the arterial phase, these lesions demonstrated marked heterogeneous enhancement, predominantly peripheral rim enhancement or internal nodular enhancement, with patchy areas of low enhancement within. On the portal venous and delayed phases, the enhancing areas progressively enlarged, exhibiting a fill-in pattern, and gradually became isodense with the surrounding splenic parenchyma. On delayed-phase images, some lesions became indistinct, with only a few lesions retaining small patchy areas of low enhancement. The multiple low-density areas in the thoracic and lumbar vertebrae and posterior elements, bilateral iliac bones, and sacrum also demonstrated patchy enhancement (**Figure 2A**), some with a fill-in pattern. The right iliac bone destruction zone and surrounding soft tissue demonstrated marked heterogeneous enhancement with a fill-in pattern (**Figure 2B**). Non-contrast MRI of the cervical, thoracic, and lumbar spine and pelvis demonstrated multiple foci of varying sizes with heterogeneous slightly prolonged T1 and iso-to prolonged T2 signal in the T7, T11, L1–4 vertebral bodies, L2 posterior elements, bilateral iliac and pubic bones, ischium, sacrum, and right femur, with high signal on short tau inversion recovery (STIR) sequences. The right iliac lesion was the largest in extent and was associated with a surrounding soft tissue mass (**Figure 3A, B**). The imaging impression was that both the splenic and multiple bone lesions were metastatic tumors.

**Figure 4:**
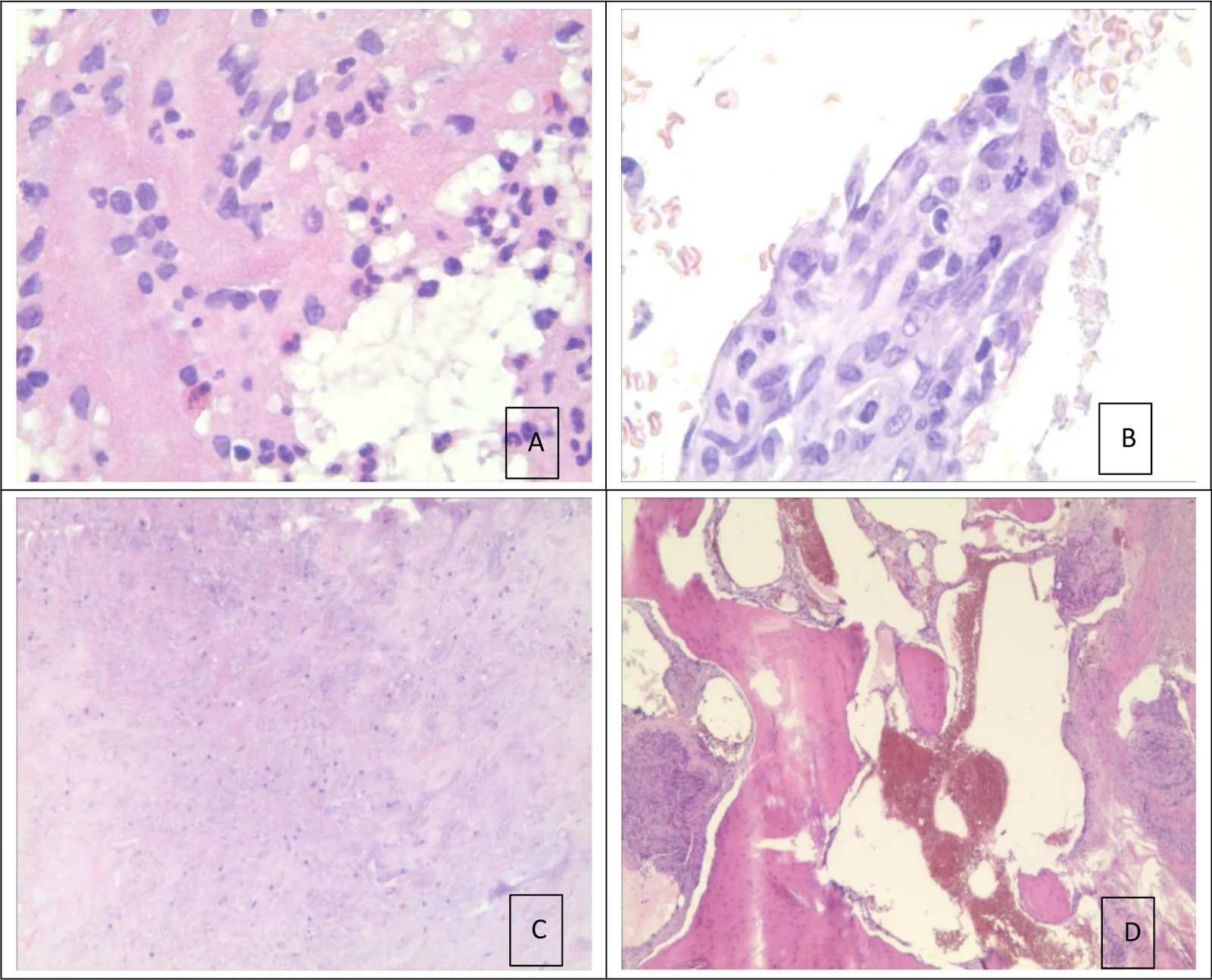
Pathological images. **(A)** Needle biopsy specimen, HE staining, ×200. Skeletal muscle tissue partially replaced by lobulated proliferating vascular tissue, with abundant infiltration of pale-staining cells. **(B)** Excision specimen, HE staining, ×200. Solid sheet-like proliferating vascular tissue; the lesional cells are epithelioid or spindle-shaped, with markedly enlarged nuclei and visible mitotic figures; the stroma shows old hemorrhage and inflammatory cell infiltration. **(C)** Excision specimen, HE staining, ×100. The tumor tissue is diffusely distributed with low cell density, and fibrous components are visible in the stroma, with a loose overall structure. **(D)** Excision specimen, HE staining, ×100. The lesion consists of a mixed proliferation of vessel-like structures of varying sizes, thicknesses, and irregular shapes, along with lobulated proliferating tissue resembling capillaries; most vessel lumina are lined by flat epithelium with no obvious atypia, and blood is present within the lumina.

**Figure 5:**
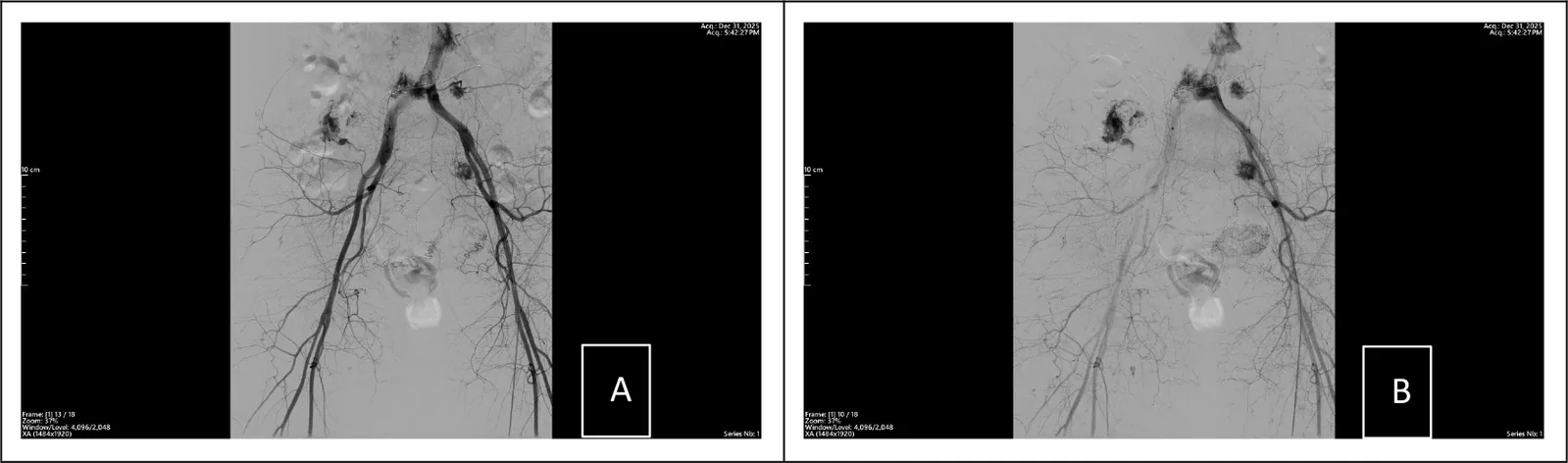
DSA angiography images. **(A)** Arterial phase, **(B)** Late arterial phase. Multiple areas of contrast blush are visible around the lumbar spine and in the bilateral pelvic regions.

To establish a tissue diagnosis, ultrasound-guided percutaneous needle biopsy of the right iliac lesion was performed. Significant intraprocedural hemorrhage was encountered. The initial pathology report described the specimen as skeletal muscle tissue partially replaced by lobulated proliferating vascular tissue, with abundant pale-staining cells (**Figure 4A**). Immunohistochemistry revealed predominantly histiocytes with some lymphocytic infiltration. The morphological and immunohistochemical findings did not reveal malignant tumor tissue. Immunohistochemical results were as follows: CD3 (sparse positive), CD20 (rare positive), CD117 (rare positive), CD34 (lobulated proliferation of small blood vessels), CD163 (partial positive), CD68 (partial positive), MPO (rare positive), Ki-67 (40% in active areas), CD30 (rare positive). Because the pathology results did not reveal malignant tumor tissue, which was discrepant with the imaging findings, the clinical team proceeded to perform open biopsy of the L2 spinous process and right iliac lesion under general anesthesia. Intraoperatively, bone destruction was identified at the L2 spinous process, and bone destruction with a bony defect was identified at the right ilium. Following tissue acquisition from the right iliac lesion, profuse bleeding from the incision was encountered, with a marked decrease in blood pressure. Compression hemostasis was ineffective. After blood transfusion and fluid resuscitation, the patient remained hypotensive, prompting emergency lower extremity digital subtraction angiography (DSA). DSA demonstrated multiple areas of contrast blush around the lumbar spine and in the bilateral pelvic regions (**Figures 5A, B**), most pronounced at the right posterior superior iliac spine. Based on the intraoperative findings, active bleeding from the right posterior superior iliac spine lesion bed was considered, and superselective angiographic embolization was performed for hemostasis.

Postoperative pathology: Gross examination—(L2 spinous process) a heap of grey-white irregular tissue measuring 4×3×1 cm, partly grey-white to grey-red and soft, partly hard. (Right ilium) a heap of grey-yellow irregular tissue measuring 4×3×1 cm, partly soft (skeletal muscle) and partly hard. Pathological diagnosis—(L2 spinous process) and (right ilium) excision specimens: The submitted material consisted of lesional tissue and destroyed bone, degenerated cartilage, collagen fibers, and skeletal muscle. The lesion was located within the medullary cavity and was composed of a mixed proliferation of dilated vessel-like structures of varying sizes, thicknesses, and irregular shapes, together with lobulated proliferating tissue resembling capillaries. The majority of the vessel lumina were lined by flat epithelium without significant atypia, and the lumina contained blood. In some areas, the tumor tissue was arranged in solid sheets; the lesional cells were epithelioid or spindle-shaped, with markedly enlarged nuclei and visible mitotic figures. Focal coagulative necrosis was present, and the stroma showed old hemorrhage and inflammatory cell infiltration. The tumor involved and destroyed the cortical bone, periosteum, and surrounding skeletal muscle (**Figures 4B-D**). Immunohistochemical results: (L2 spinous process) CD34 (partial positive), ERG (positive), Ki-67 (10%); (Right ilium) lesional tissue within skeletal muscle: CD34 (positive), ERG (positive), Ki-67 (20% in active areas). (Right ilium) solid sheet-like lesional area within bone: CD31 (positive), SMA (rare positive), Fli-1 (positive), P53 (partial positive, variable intensity, suggesting wild-type), MDM2 (negative), SATB2 (negative). Pathological diagnosis: The morphological and immunohistochemical findings supported a vascular-origin neoplasm; in conjunction with the clinical findings (multiple lesions in the axial skeleton and spleen), the findings were consistent with a mixed-type vascular neoplasm with partially well-differentiated angiosarcomatous changes.

**Figure 6:**
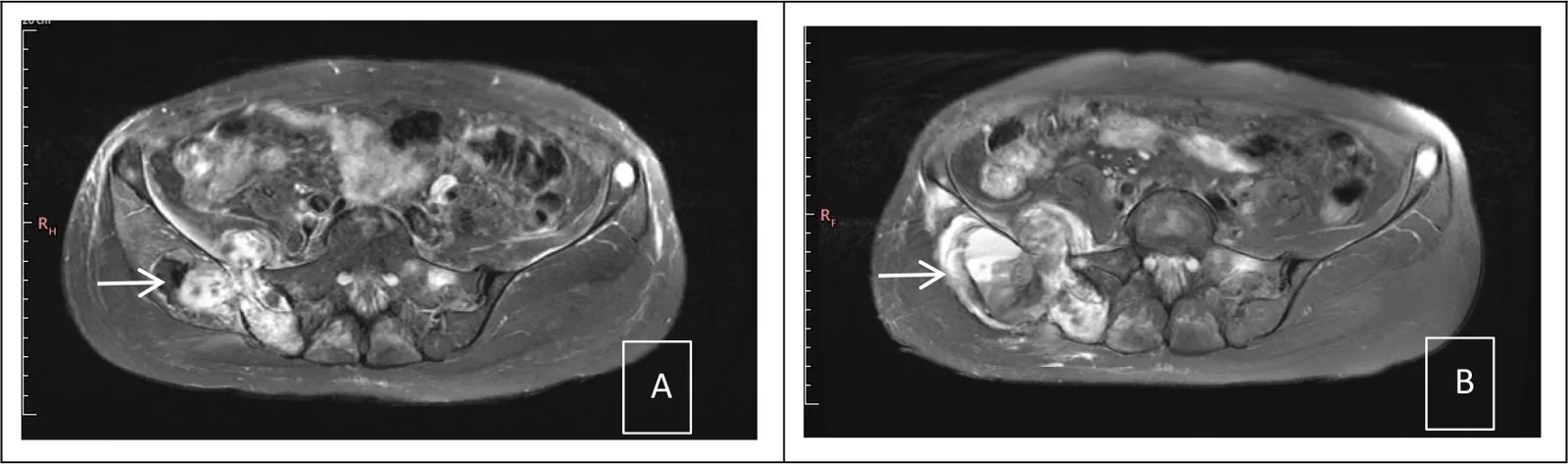
Comparison of initial and follow-up MRI. Axial STIR images of the pelvis. **(A)** Image at admission shows bone destruction and a surrounding soft tissue mass in the right ilium (arrows). **(B)** Image from the 21-day follow-up shows interval enlargement of the lesion (arrows).

Twenty-one days after admission, follow-up pelvic MRI was performed, demonstrating abnormal signal in the sacrum, bilateral iliac bones, pubic bones, ischium, and right femur, with some lesions showing slight interval enlargement compared with the prior examination, with the largest axial dimension of the right iliac lesion increasing from 89.7 mm×65.4 mm to 100.7 mm×78.6 mm (**Figures 6A, B**).

After pathological confirmation, given the extensive bone metastases, the family requested discharge. The patient was advised to continue active symptomatic and supportive treatment after discharge and to seek further treatment at a higher-level hospital. A telephone follow-up 3 months after discharge revealed that the patient received only symptomatic treatment after discharge and passed away approximately 41 days after discharge.

## 3 Discussion

### 3.1 Determination of the primary site and clinical diagnosis

The patient presented with simultaneous splenic and skeletal lesions, necessitating consideration of four differential diagnostic scenarios: dual primary tumors, dual metastatic tumors (i.e., metastases from a third unknown primary to both organs), primary splenic tumor with bone metastasis, and primary bone tumor with splenic metastasis. Accurate differentiation is of critical importance for tumor staging and treatment planning. Although the possibilities of multi-primary angiosarcoma involving the spleen and bones, as well as metastases to both the spleen and bones from an occult angiosarcoma, cannot be completely ruled out, based on the following evidence, we favor the diagnosis of primary splenic angiosarcoma with bone metastases:(1) Primary angiosarcoma of bone is exceedingly rare, accounting for less than 1% of all primary bone tumors (1). When bone angiosarcoma does occur, it typically presents as a solitary lesion rather than widespread polyostotic involvement (10). In contrast, PSA is well-documented for its propensity for early and widespread metastasis, with reported metastatic rates of 70–85% (3, 4), and bone is among the common metastatic sites (5, 6, 11). The clinical presentation in our case is more consistent with the metastatic pattern of PSA. Furthermore, whole-body screening did not identify any other primary malignancy, which argues against metastatic carcinoma with dual-organ involvement. (2) The histological features of the multiple bone lesions were concordant, both demonstrating a mixed-type vascular neoplasm with well-differentiated angiosarcomatous components. The immunohistochemical profile (CD31+, CD34+, ERG+, Fli-1+) was consistent across all specimens, confirming a common endothelial lineage. Importantly, the bone lesions did not exhibit any features suggestive of a primary bone tumor (e.g., osteoid production, chondroid matrix, or small round blue cell morphology). Negative MDM2 and SATB2 staining further excluded dedifferentiated liposarcoma and osteosarcoma, respectively. The morphological and immunophenotypic identity of the lesions at both sites supports a clonal relationship, consistent with metastatic spread from a single primary tumor. (3) On contrast-enhanced CT, the splenic lesions demonstrated a characteristic enhancement pattern: peripheral nodular enhancement in the arterial phase with progressive centripetal fill-in during the portal venous and delayed phases. The bone lesions similarly demonstrated patchy and fill-in enhancement patterns. The identical enhancement behavior across both splenic and skeletal sites supports a unified pathological process rather than independent primary tumors. This is further supported by the literature demonstrating that PSA metastases to bone retain the vascular enhancement characteristics of the primary tumor (12, 13). (4) Multiple case reports and case series have documented that PSA commonly presents with bone marrow involvement as the initial manifestation. Kmail et al. (5) reported a case of primary splenic angiosarcoma presenting with unexplained anemia in a young adult. Wu et al. (6) described a case of metastatic PSA presenting with anemia and bone marrow fibrosis. Misiak et al. (11) reported pancytopenia related to splenic angiosarcoma with bone marrow metastasis. These cases collectively demonstrate that bone involvement in the context of PSA is typically metastatic rather than a second primary. Furthermore, PSA has been diagnosed on bone marrow biopsy (2), further underscoring the predilection of this tumor for skeletal dissemination.

The limitation of this case is that the splenic lesion did not obtain histopathological confirmation. The diagnosis of primary splenic angiosarcoma is a presumptive diagnosis based on the high consistency between the characteristic imaging findings and the angiosarcoma pathology of the bone metastatic lesions. Other entities that may cause multiple osteolytic lesions were considered and excluded: (1) Multiple myeloma: The patient had no M-protein, no Bence-Jones proteinuria, and no hypercalcemia, and the pathological morphology of the bone lesions was not supportive. (2) Lymphoma: The pathological examination of the bone lesions revealed no lymphocytic infiltration, immunohistochemistry showed no LCA positivity, and the enhancement pattern of the splenic lesions on contrast-enhanced CT was more consistent with a vascular tumor rather than lymphoma.

### 3.2 Histopathological characteristics of angiosarcoma and the discordance between differentiation and biological behavior

The 2020 World Health Organization classification defines angiosarcoma as a malignant neoplasm demonstrating endothelial differentiation, either morphologically or immunophenotypically (7). Histologically, angiosarcoma exhibits a broad morphological spectrum ranging from well-differentiated to poorly differentiated forms.

Well-differentiated angiosarcoma is characterized by irregularly shaped, anastomosing vascular channels lined by relatively bland endothelial cells with minimal cytological atypia. These vessels may infiltrate surrounding tissues in an infiltrative pattern that can closely mimic benign vascular lesions such as hemangioma (8). Mitotic figures are sparse, and necrosis is typically absent. The key distinguishing features include the infiltrative growth pattern, with vessels dissecting through normal tissue structures, and the presence of irregularly shaped, anastomosing channels rather than the well-circumscribed lobular architecture of hemangioma. Moderately differentiated angiosarcoma exhibits more conspicuous endothelial cell atypia, with nuclear enlargement, hyperchromasia, and occasional mitotic figures. Solid areas may be present but are limited in extent. Poorly differentiated angiosarcoma demonstrates marked cellular pleomorphism, frequent mitoses, and extensive solid growth with epithelioid or spindle cell morphology. Necrosis is common, and vascular architecture may be barely recognizable, requiring immunohistochemical confirmation with endothelial markers. The immunohistochemical profile of angiosarcoma includes consistent positivity for endothelial markers: CD31 (the most sensitive and specific), ERG (nuclear marker, highly specific), CD34 (sensitive but less specific), and Fli-1 (nuclear marker). The Ki-67 proliferation index varies widely but is typically elevated in poorly differentiated tumors.

A pivotal observation in the present case is the remarkable discrepancy between the high-grade histological appearance and the extraordinarily aggressive clinical course. The tumors at both the L2 spinous process and right ilium showed heterogeneity — characterized by the coexistence of mostly well-differentiated vessel-forming areas, solid areas exhibiting obvious cellular atypia, epithelioid hyperplastic areas, and focal necrosis, consistent with features of high-grade angiosarcoma. The Ki-67 proliferation indices of the L2 and iliac lesions were 10% and 20%, respectively, indicating relatively low/moderate proliferative activity. Yet, the tumor had already produced multiple bone metastases, and follow-up MRI performed only 21 days after the initial scan demonstrated imaging-visible interval enlargement of the lesions.

This discordance is not unique to our case. Xu et al. (8) reported a case of well-differentiated angiosarcoma of the spleen that closely mimicked hemangioma on imaging but exhibited aggressive clinical behavior, including local recurrence and distant metastasis. Xie et al. (9) reported similar findings, noting that well-differentiated splenic angiosarcoma may show no obvious abnormality on PET/CT yet demonstrate a strong metastatic propensity, and further noted that a low Ki-67 index does not preclude an aggressive clinical course. The underlying mechanism for this phenomenon remains incompletely elucidated and may be related to multiple factors: First, the heterogeneous nature of angiosarcoma is well-recognized (7, 13). A single tumor may contain areas of varying differentiation, and sampling limitations—particularly with fine-needle biopsy specimens—may underestimate the true biological potential. In our case, the initial needle biopsy completely failed to detect the malignant component, a phenomenon that has been documented in vascular tumors (8). Even the surgical specimens showed focal high-grade areas, suggesting that the aggressive behavior may be driven by small subclones that are not adequately represented in the overall histological assessment. Second, recent molecular studies have revealed that the genomic landscape of angiosarcoma is highly heterogeneous (14, 15). Recurrent genetic alterations include MYC amplification (particularly in radiation-associated and lymphedema-associated angiosarcoma), FLT1 amplification, KDR mutations, and TP53 mutations. Importantly, these molecular alterations may be present even in histologically well-differentiated tumors and can drive aggressive biological behavior independent of morphological grade. The MYC-amplified subset of angiosarcoma, in particular, has been associated with aggressive clinical behavior regardless of histological differentiation (15). Third, the vascular nature of the tumor itself contributes to its aggressive behavior. Angiosarcoma arises from endothelial cells that possess intrinsic capabilities for invasion, migration, and angiogenesis. Even well-differentiated tumor cells retain these properties, enabling hematogenous dissemination at an early stage (16). This biological characteristic may explain the rapid disease progression observed in our case despite the relatively low Ki-67 index.

The clinical implication of this discordance is profound: pathologists and clinicians should not be reassured by a well-differentiated appearance in vascular tumors. When clinical and imaging features suggest aggressive disease, the pathological diagnosis should be interpreted with caution, and repeat or more extensive tissue sampling may be warranted.

### 3.3 Imaging features and differential diagnosis of angiosarcoma

On non-contrast CT, splenic angiosarcoma typically presents as multiple low-density nodules within an enlarged or normal-sized spleen (17, 18). The lesions may protrude beyond the splenic contour, and areas of hemorrhage or necrosis may be present. The hallmark finding on contrast-enhanced CT is progressive centripetal enhancement: the arterial phase shows peripheral nodular or rim enhancement, with gradual fill-in during the portal venous and delayed phases, eventually approaching the attenuation of normal splenic parenchyma (17). This pattern closely mimics that of hemangioma, creating a significant diagnostic pitfall. On MRI, splenic angiosarcoma typically appears as T1-hypointense and T2-hyperintense lesions with heterogeneous internal architecture. Contrast-enhanced MRI demonstrates enhancement patterns similar to those seen on CT. The presence of hemorrhagic components, necrotic areas, and signal heterogeneity are features that favor angiosarcoma over hemangioma (12).

On ultrasound, splenic angiosarcoma typically manifests as multiple hypoechoic or heterogeneously hypoechoic nodules with ill-defined margins; due to intratumoral hemorrhage and necrosis, complex mixed echogenicity or a mosaic pattern may occasionally be observed (12, 13, 17). Abundant color Doppler signals can be detected within the tumor, with low-resistance arterial waveforms, indicating the presence of rich but structurally abnormal tumor neovascularization lacking normal vasomotor regulation (9, 12, 13). On contrast-enhanced ultrasound (CEUS), angiosarcoma typically demonstrates heterogeneous enhancement in the arterial phase, with chaotic and irregular filling patterns. In the late portal venous and parenchymal phases, washout or persistent heterogeneous hypo-enhancement is commonly observed (13).

Osseous metastases from angiosarcoma typically present as osteolytic lesions with variable soft tissue components. On MRI, these lesions demonstrate T1-hypointense and T2-heterogeneous signal with STIR hyperintensity. The enhancement pattern mirrors that of the primary tumor, showing progressive fill-in. The bone lesions in our case demonstrated exactly this pattern.

The most challenging and clinically consequential differential diagnosis is between angiosarcoma and hemangioma, both of which are vascular neoplasms with overlapping imaging features (**Table 1**).

**Table 1:** Typical imaging features of angiosarcoma and hemangioma.

Feature	Angiosarcoma	Hemangioma
Number	Often multiple	Usually solitary
Margin	Ill-defined, infiltrative	Well-circumscribed
Contour deformity	May protrude beyond organ cap-sule	Confined within organ
Internal architecture	Heterogeneous, necro-sis/hemorrhage	Homogeneous
US echogenicity	Hypoechoic,heterogeneous, “mo-saic” pattern	Hyperechoic, homogeneous
Color Doppler US	Internal flow, low-resistance ar-teries	Minimal/absent internal flow
CEUS	Heterogeneous enhancement, washout	Nodular fill-in, sustained en-hancement
Arterial phase (CT/MRI)	Peripheral nodular enhancement, heterogeneous	Peripheral nodular enhancement, homogeneous
Portal/delayed phase (CT/MRI)	Progressive fill-in, may not match parenchyma	Complete fill-in, matches parenchyma
Unenhanced areas (CT/MRI)	Present (necrosis, hemorrhage)	Absent
Adjacent tissue invasion	Present	Absent
Growth rate	Rapid	Slow or stable

Koyama et al. (17) emphasized that early CT case reports of hepatic angiosarcoma mimicking hemangioma could likely be attributed to single-phase imaging, often during the delayed phase when fill-in is most pronounced. Multiphasic imaging with careful evaluation of all phases is essential for accurate differentiation. The presence of any atypical features—heterogeneity, necrosis, incomplete fill-in, capsular breach, or rapid growth—should raise suspicion for angiosarcoma even when the overall enhancement pattern resembles hemangioma. The differential diagnosis also includes epithelioid hemangioendothelioma (EHE), which similarly demonstrates vascular enhancement but typically has a more indolent clinical course and characteristic myxohyaline stroma, and Kaposi sarcoma, which occurs in specific clinical contexts such as HIV infection or immunosuppression.

### 3.4 Clinical risks: hemorrhage following biopsy of vascular tumors

This case vividly illustrates a critical and potentially life-threatening risk in the management of vascular tumors: intractable hemorrhage following biopsy. Both the initial needle biopsy and the subsequent open biopsy were complicated by significant bleeding, with the open biopsy precipitating hemodynamic instability requiring emergency embolization.

Angiosarcoma is a tumor of endothelial origin that forms aberrant, poorly organized vascular channels. These neoplastic vessels lack the normal muscularis and adventitial layers of physiological blood vessels, and their walls are often composed of only a single layer of endothelial cells—sometimes with cytological atypia—directly exposed to the lumen (7, 14). This structural fragility renders the tumor inherently prone to spontaneous and iatrogenic hemorrhage. Additionally, the rich vascularity of the tumor, combined with its infiltrative growth pattern that disrupts normal tissue architecture, means that any biopsy tract inevitably encounters fragile neovascular structures. In bone lesions specifically, the risk is further compounded by several factors: (1) the medullary cavity contains an abundant vascular supply that cannot be easily compressed; (2) cortical bone destruction eliminates the structural constraint that would otherwise contain hemorrhage; and (3) the associated soft tissue mass typically contains additional networks of tumor vessels lacking surrounding connective tissue support. In our case, DSA angiography demonstrated marked tumor blush in the late arterial phase, confirming the rich vascularity of the tumor.

Gao et al. (19) reported a case of angiosarcoma with skull masses in which biopsy of the calvarial lesion was complicated by significant hemorrhage. The authors emphasized that the vascular nature of the tumor, combined with the coagulopathy that frequently accompanies angiosarcoma (due to consumptive coagulopathy or bone marrow infiltration), creates a particularly hazardous situation for invasive procedures. Mazzocchi et al. (20) reported a case of angiosarcoma of the breast associated with Kasabach-Merritt syndrome, a consumptive coagulopathy characterized by thrombocytopenia and life-threatening hemorrhage, further underscoring the intrinsic hemorrhagic tendency of these tumors. The broader literature on vascular tumor biopsy supports these observations. When imaging features suggest a vascular tumor, pre-biopsy angiographic assessment should be considered, and biopsy should be performed with adequate hemostatic measures available (19, 20).This recommendation requires verification by more studies. In our case, if the vascular nature of the tumor had been identified on imaging prior to biopsy, pre-biopsy angiographic assessment and possible prophylactic embolization might have mitigated the life-threatening hemorrhagic event that ultimately occurred.

## 4 Conclusion

We report a rare case of suspected primary splenic angiosarcoma presenting with multiple bone destruction as the initial manifestation. This case reveals several key clinical lessons: First, when splenic and skeletal lesions coexist, the rarity of primary bone angiosarcoma and the propensity of PSA for early and widespread metastasis strongly favor PSA with bone metastases. Second, the histological differentiation of angiosarcoma may be significantly discordant with its biological behavior: high-grade tumors, despite their benign-appearing morphology, may pursue an aggressive clinical course. Third, the imaging features of angiosarcoma can closely mimic those of benign vascular tumors, and careful attention to distinguishing features—including lesion heterogeneity, necrosis, incomplete fill-in, capsular breach, and rapid growth—is essential for accurate diagnosis. Fourth, biopsy of vascular tumors, particularly those involving bone, carries a substantial risk of intractable hemorrhage. Pre-procedural angiographic assessment, proactive hemostatic preparedness, and multidisciplinary coordination are crucial to mitigate this potentially life-threatening complication.

## Data Availability

The original contributions presented in the study are included in the article/supplementary material. Further inquiries can be directed to the corresponding author.
